# Normative Laboratory Value Ranges in Pediatric Patients Who Underwent Evaluation for MIS-C

**DOI:** 10.1155/emmi/2660909

**Published:** 2025-11-25

**Authors:** Thomas Graf, Nathan M. Money, Sindhoosha Malay, Nori Minich, Risa Bochner, Bari Winik, Ron Kaplan, Jennifer Y. Colgan, Erin Aldag, Ryan O' Sullivan, Guliz Erdem, Allayne Stephans

**Affiliations:** ^1^Department of Pediatrics, Division of Pediatric Hospital Medicine, Rainbow Babies and Children's Hospital, Case Western Reserve University, Cleveland, Ohio, USA; ^2^Department of Pediatrics, Division of Pediatric Hospital Medicine, University of Utah School of Medicine, Salt Lake City, Utah, USA; ^3^Department of Pediatrics, New York City Health and Hospitals Harlem Hospital, Columbia University College of Physicians and Surgeons, New York, New York, USA; ^4^Department of Pediatrics, Division of Pediatric Hospital Medicine, Cohen Children's Medical Center-Northwell Health and Zucker School of Medicine at Hofstra/Northwell, New Hyde Park, New York, USA; ^5^Department of Pediatrics, Seattle Children's Hospital, University of Washington School of Medicine, Seattle, Washington, USA; ^6^Department of Pediatrics, Division of Pediatric Emergency Medicine, Northwestern University Feinberg School of Medicine, Chicago, Illinois, USA; ^7^Department of Pediatrics, Children's Hospital of Philadelphia, Philadelphia, Pennsylvania, USA; ^8^Division of Infectious Diseases, Department of Pediatrics, Nationwide Children's Hospital, Columbus, Ohio, USA

## Abstract

**Background and Objectives:**

Diagnosis of multisystem inflammatory syndrome in children (MIS-C) is challenging due to symptom overlap with other common conditions. The primary objective of this multicenter study was to establish normative laboratory values in MIS-C patients and compare to patients evaluated for MIS-C who had other final diagnoses.

**Methods:**

Five hospitals reviewed records of a cohort of patients 6 months to 18 years old who were evaluated for MIS-C between March 31^st^ 2020 and February 1^st^ 2022. Patient demographics, clinical presentation, and laboratory values were compared in patients with a final diagnosis of MIS-C versus all other conditions. Patients' final diagnoses and laboratory values for each diagnosis were reported.

**Results:**

Of the 1319 patients that were evaluated for MIS-C at presentation, 293 (22.2%) received a final diagnosis of MIS-C. MIS-C patients had statistically significant differences in laboratory values including D-dimer, ferritin, fibrinogen, troponin, and brain natriuretic peptide when compared to patients evaluated for MIS-C but subsequently diagnosed with other conditions. The most common non-MIS-C diagnoses within this cohort were viral illnesses (26.7%), acute COVID-19 (12.0%), pyelonephritis (6.4%), fever of unknown origin (4.9%), pneumonia (4.7%), gastroenteritis (4.7%), Kawasaki disease (3.7%), other bacterial infections, (3.1%), rheumatologic conditions (3.1%), and sepsis (2.8%).

**Conclusions:**

Many patients who underwent laboratory evaluation for MIS-C during the COVID-19 pandemic received a final diagnosis other than MIS-C. These data may aid pediatricians in differentiating between MIS-C and other febrile conditions as well as reduce unnecessary testing.

## 1. Background

Multisystem inflammatory syndrome in children (MIS-C) is an inflammatory condition associated with previous infection with SARS-CoV-2 that was first described in 2020 [1–3]. The diagnosis of MIS-C is challenging as there are no specific clinical or laboratory tests that differentiate it from other febrile conditions of childhood. MIS-C occurs between 3 and 6 weeks after SARS-CoV-2 infection and is characterized by fevers and systemic signs of inflammation, most commonly involving the integumentary, gastrointestinal, and cardiac systems [4, 5]. The Centers for Disease Control and Prevention (CDC) criteria require laboratory proof of a SARS-CoV-2 infection, and probable cases require meeting clinical criteria and linkage to a SARS-CoV-2 exposure if laboratory testing is unavailable or negative [6]. Many patients initially evaluated for MIS-C are reported to have other diagnoses, including Kawasaki disease, acute COVID-19, other viral and bacterial infections, and rheumatologic conditions [7, 8].

Laboratory abnormalities reported in MIS-C typically include neutrophilia, lymphopenia, thrombocytopenia, hypoalbuminemia, and elevations in inflammatory markers and acute phase reactants [9, 10]. D-dimer was noted to be particularly elevated in MIS-C patients by some studies, though the significance of this finding is unknown as D-dimer is infrequently used in the evaluation of pediatric patients [11, 12]. Similarly, the normative ranges of common laboratory tests typically obtained for MIS-C evaluation are not well established in other pediatric conditions. Most of the pediatric literature on laboratory markers in MIS-C reported values exclusively in MIS-C patients or in comparison to a small number of conditions, primarily acute COVID-19 and Kawasaki disease [13–15]. Establishing typical laboratory value ranges for febrile conditions in children may aid providers in the diagnosis and management of this cohort. This may also reduce unnecessary testing in children for whom MIS-C is unlikely.

The primary objective of this study was to compare normative laboratory value ranges for children with MIS-C to those evaluated for MIS-C but subsequently diagnosed with other conditions. Secondary objectives were to identify the spectrum of final diagnoses in patients evaluated for MIS-C and to describe normative laboratory ranges for these pediatric diagnoses.

## 2. Methods

### 2.1. Study Population

Five pediatric hospitals in the United States performed a multicenter retrospective cohort analysis of patients evaluated for MIS-C. To identify patients evaluated for MIS-C, electronic medical records at each site were queried for encounters of patients aged 6 months to 18 years old in which a D-dimer or SARS-CoV-2 IgG antibody test was sent from the period of March 3﻿1^st^ 2020 to February1^st^ 2022. Patients were excluded if SARS-CoV-2 IgG or D-dimer was sent without medical documentation of suspected acute COVID-19 infection or MIS-C. The Institutional Review Board (IRB) at each of these five institutions approved this study prior to data collection. The IRB approval numbers for each institution are the following: STUDY20210064 (University Hospitals), 00003192 (Seattle Children's Hospital), 00001932 (Nationwide Children's Hospital), IRB_00142644 (Primary Children's Hospital), and IRB 2021-4709 (Lurie Children's Hospital).

### 2.2. Data Collection

Demographic information, duration and nature of symptoms, past medical history, vital signs at presentation, medications received, laboratory testing and results, imaging tests and results, emergency department disposition, and final diagnoses were collected through data query and manual chart review. Study data were collected and managed using REDCap electronic data capture tools hosted at University Hospitals (with the support of the Clinical and Translational Science Award [CTSC]–UM1TR004528) [16, 17]. Laboratory tests included D-dimer, fibrinogen, partial thromboplastin time (PTT), prothrombin time (PT), international normalized ratio (INR), lactate dehydrogenase (LDH), albumin, ferritin, troponin, brain natriuretic protein (BNP), SARS-CoV-2 polymerase chain reaction (PCR), white blood cell count (WBC), absolute neutrophil count (ANC), absolute lymphocyte count (ALC), hemoglobin, platelet count, erythrocyte sedimentation rate (ESR), C-reactive protein (CRP), procalcitonin, aspartate aminotransferase (AST), alanine aminotransferase (ALT), antinuclear antibody (ANA), interleukin-6 (IL-6), triglyceride level, urinalysis, and blood and urine cultures. For each laboratory test, the first value obtained during the hospital encounter was recorded. If the patient received repeat testing, the maximum and minimum values were also recorded. Final discharge diagnoses were confirmed by manual chart reviews by the study investigators. Discharge diagnoses were identified through final discharge diagnosis coding as well as discharge summary documentation for the encounter. MIS-C diagnosis was confirmed by clinicians using the CDC criteria that were available at the time [6]. Probable cases of MIS-C as determined by clinicians were included as MIS-C cases by the study investigators. Our data collection tool allowed for multiple final diagnoses to be assigned to a single patient. However in the case of patients with MIS-C, only the final diagnosis of MIS-C was assigned. This was also true for patient assigned a diagnosis of fever of unknown origin. Data were reviewed for accuracy and completeness by the lead study investigators.

### 2.3. Data Analysis

Patients were grouped into those who had a final discharge diagnosis of MIS-C versus patients with other final diagnoses. Demographic characteristics were described for patients with and without a diagnosis of MIS-C. The frequency of final diagnoses other than MIS-C was also identified. Clinical characteristics of the patients were described using median and interquartile range (25th percentile, 75th percentile) for continuous variables and frequency and percentages for categorical variables, as appropriate. Categorical variables were analyzed using a chi-square or Fisher's exact test. Continuous variables were assessed by the Mann–Whitney *U* Test. A *p* value of less than 0.05 was considered statistically significant for the analysis. Analyses were performed using IBM SPSS Statistics for Windows, Version 29.0 (IBM Corp., Armonk, NY, USA), or SAS, Version 9.4 (SAS Institute Inc., Cary, NC, USA).

## 3. Results

A total of 2182 patients were identified over the course of the study period and underwent chart review. 1319 patients met inclusion criteria. Of these, 293 (22.2%) were diagnosed with MIS-C, and 1026 (77.8%) received an alternative final diagnosis (Table 1). Children diagnosed with MIS-C were more likely to be male (65.5% vs. 49.3%). The median age was higher in patients with MIS-C (9.0 years, IQR 5.0–12.0 vs. 6.0 years, IQR 2.0–13.0). Patients with MIS-C were more likely to be admitted (98.0% vs. 70.9%), had longer lengths of stay (5.0 days, IQR 4.0–7.0 vs. 3.0 days, IQR 1.7–5.0), and were more likely to require ICU level care (54.9% vs. 15.6%). All *p* values < 0.001.

**Figure 1:**
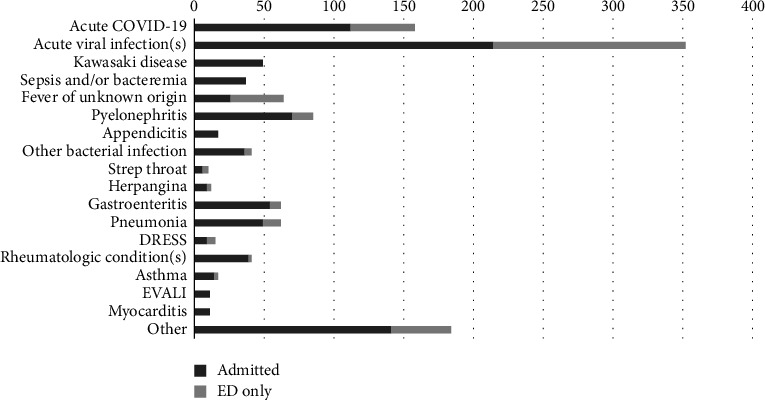
Other final diagnoses in patients evaluated for MIS-C. DRESS—drug reaction with eosinophilia and systemic symptoms; EVALI—e-cigarette or vaping use–associated lung injury.

The laboratory values for D-dimer (*p* < 0.001), fibrinogen (*p* < 0.001), ferritin (*p* < 0.001), BNP (*p* < 0.001), PT (*p*=0.004), INR (*p* < 0.001), ANC (*p* < 0.001), ESR (*p* < 0.001), CRP (*p* < 0.001), procalcitonin (*p* < 0.001), AST (*p* < 0.006), ALT (*p* < 0.001), IL-6 (*p*=0.002), and triglycerides (*p*=0.017) were significantly higher in patients with MIS-C versus those who received other diagnoses (Table 2). Albumin (*p* < 0.001), ALC (*p* < 0.001), hemoglobin (*p* < 0.001), and platelets (*p* < 0.001) were lower in MIS-C patients. Troponin levels were elevated in 37.5% of MIS-C patients versus 8.3% (*p* < 0.001) of patients with other diagnoses (Supporting Table 1). The most common diagnoses in patients evaluated for MIS-C who received alternative diagnoses included viral illnesses (i.e., adenovirus, rhinovirus, Epstein–Barr virus, etc.) (26.7%), acute COVID-19 (12.0%), pyelonephritis (6.4%), fever of unknown origin (4.9%), pneumonia (4.7%), gastroenteritis (4.7%), Kawasaki disease (3.7%), other bacterial infections (3.1%), other rheumatologic conditions (3.1%), and sepsis (2.8%) (Figure 1).

**Figure 2:**
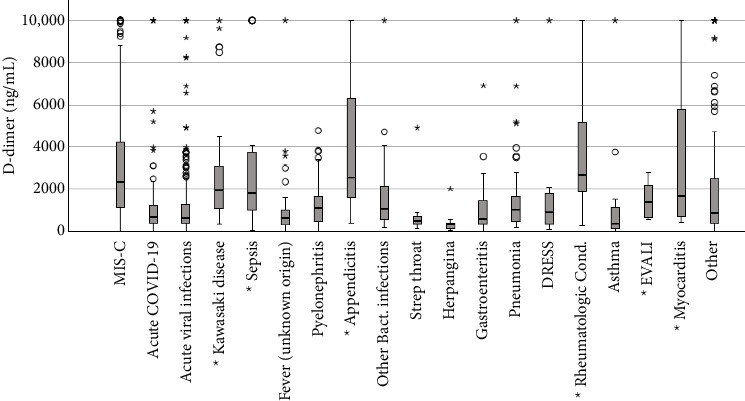
Plot of interquartile ranges of D-dimer for MIS-C and individual other diagnoses. Symbol ^∗^ on *X*-axis next to disease condition indicates a range that is not significantly different from MIS-C, according to Mann–Whitney *U* test. MIS-C—multisystem inflammatory syndrome in children; EVALI—e-cigarette or vaping use–associated lung injury.

Median laboratory values and IQR were also compared between MIS-C diagnoses and other individual diagnoses (Table 3). There were several laboratory tests that had notable significant differences between MIS-C and the most common other conditions identified. The lower IQR (229 mcg/L) of ferritin in MIS-C was higher than the median ferritin in most other conditions. BNP levels in MIS-C tended to have a higher median than most other diagnoses (312 pg/mL, IQR 89–1223). MIS-C cases had an ALC with a lower IQR (1100 cells/μL, IQR 700–1721) than most other conditions except rheumatologic conditions (1230 cells/μL, IQR 800–3018) and sepsis (1400 cells/μL, IQR 685–2280). The IQR of platelet count in MIS-C (174 × 10^3^/μL, IQR 125–237) was statistically different from all other reported conditions except sepsis (198 × 10^3^/μL, IQR 126–291). MIS-C cases also had significant differences in the IQR of inflammatory markers. MIS-C cases had a statistically higher IQR for CRP (13.8 mg/dL, IQR 7.5–21.2) than all other conditions except sepsis (10.0 mg/dL, IQR 4.2–21.8). Procalcitonin was also elevated in MIS-C with an IQR (2.57 ng/mL, IQR 1.03–8.69) higher than most other conditions and similar to sepsis (3.41 ng/mL, IQR 1.18–48.86). Several conditions had elevated D-dimer levels with interquartile ranges that overlap with MIS-C such as Kawasaki disease (1965 ng/mL, IQR 1100–3148), sepsis (1826 ng/mL, IQR 1018–3783), and rheumatologic conditions (2670 ng/mL, IQR 1835–5205) (Figure 2).

## 4. Discussion

This study presents findings from a large, multicenter cohort of patients who underwent evaluation for MIS-C comparing laboratory data in patients diagnosed with MIS-C to those diagnosed with other conditions. The most notable and clinically significant laboratory changes among MIS-C patients were D-dimer, albumin, fibrinogen, ferritin, BNP, troponin, ALC, CRP, ESR, procalcitonin, and platelets. D-dimer, ferritin, fibrinogen, and BNP were elevated in most MIS-C cases compared to non-MIS-C cases. There was a relative decrease in MIS-C cases in the levels of albumin, ALC, and platelets. MIS-C patients also tended to have higher median CRP, ESR, and procalcitonin levels. Interestingly, procalcitonin was elevated in MIS-C as compared to most other conditions but had an overlapping IQR with sepsis. Furthermore, this study can inform clinicians ofthe laboratory tests that are not useful in diagnosing MIS-C. While coagulation studies, IL-6, and triglycerides had statistically significant differences between MIS-C and other diagnoses, the change in median values would not likely be meaningful to clinicians for diagnostic purposes.

Although no single test was individually specific, done collectively these laboratory tests could be useful in distinguishing MIS-C from other clinical conditions. MIS-C cases tended to have elevations in the inflammatory markers CRP, ESR, and procalcitonin with a D-dimer value greater than 1000 and ferritin value greater than 200 in combination with an abnormal troponin and BNP. Cardiac involvement in the form of elevations of troponin and BNP was observed much more frequently in MIS-C than in other conditions reported in this study, with the notable exception of myocarditis. Thus, elevated cardiac markers in the presence of elevations of multiple inflammatory markers and acute phase reactants are suggestive of MIS-C.

We identified a heterogeneous group of final diagnoses in patients who were initially evaluated for MIS-C. This list of conditions that can mimic the presenting symptoms of MIS-C ranged from viral infections, sepsis, pyelonephritis, pneumonia, gastroenteritis, myocarditis, e-cigarette or vaping use–associated lung injury (EVALI), and drug reaction with eosinophilia and systemic symptoms (DRESS). The study period was during a time in which the number of MIS-C cases was relatively high, and unsurprisingly clinicians had a low threshold to include MIS-C in the differential diagnosis.

This is one of very few studies that include the spectrum of diagnoses that underwent initial evaluation for MIS-C. Many of the previous studies performed compared laboratory values in MIS-C to COVID-19, Kawasaki disease, and macrophage activation syndrome (MAS). In these studies, patients with MIS-C had higher CRP, D-dimer, and ANC as well as lower ALC and platelets compared to Kawasaki disease and COVID-19 [18–20]. Patients with MIS-C had lower ferritin levels than in patients with sepsis and MAS [21, 22]. Our findings are similar to these studies. Other case series of MIS-C patients also produced similar laboratory findings to this study with regard to WBC, ALC, platelet count, fibrinogen, CRP, ESR, procalcitonin, troponin, and BNP in MIS-C cases [23, 24]. We found that D-dimer and ferritin were generally higher in MIS-C than in cases of COVID-19 and Kawasaki disease but were similarly elevated in rheumatologic conditions. CRP was higher and platelet count was lower in MIS-C than these other conditions. The laboratory ranges that we report for MIS-C, COVID-19, and Kawasaki disease are similar to ranges previously reported in the literature [25, 26].

Many of the alternate final diagnoses also had abnormal median and ranges of laboratory test results. This study provides ranges of values of laboratory tests for a spectrum of common pediatric conditions. D-dimer was frequently used as part of screening laboratory tests for MIS-C, and although children with MIS-C had higher median D-dimer levels than the other final diagnoses, there were several pediatric diagnoses in this study that also had markedly elevated D-dimer levels. Viral infections, acute COVID-19 infection, otitis media, pyelonephritis, pneumonia, sepsis, myocarditis, rheumatologic conditions, and Kawasaki disease all had significant elevations. These laboratory ranges can provide a primer to further validate the normative values in these conditions.

### 4.1. Limitations

This study has several limitations due to its retrospective design. One limitation is that the number of patients with any one alternative diagnosis was relatively small, which limited our ability to compare laboratory findings between these diagnoses. The grouping of rheumatologic diseases and viral infections further limited our ability to compare specific diagnoses within these categories. It is also possible that some final diagnoses were incorrectly categorized during the chart review process. Additionally, we allowed multiple diagnoses per patient aside from MIS-C and fever of unknown origin. This could lead to overlap of laboratory values due to more than one diagnosis being represented by a single patient.

## 5. Conclusions

Many pediatric patients who presented with fever and nonspecific symptoms during the COVID-19 pandemic underwent laboratory evaluation for MIS-C, though most of these patients were diagnosed with a condition other than MIS-C. MIS-C patients had significantly different laboratory values when compared to patients evaluated for MIS-C but subsequently diagnosed with other conditions. In particular, elevations in BNP and troponin were more common in MIS-C than in most other conditions except myocarditis. Our data may be useful to help clinicians attempting to differentiate MIS-C from other conditions and decrease unnecessary testing in those whom MIS-C is unlikely. This study also provides suggestive ranges for laboratory tests that are infrequently sent in pediatric conditions other than MIS-C.

## Figures and Tables

**Table 1:** Demographic information of patients evaluated for MIS-C.

Characteristics	Overall *n* = 1319^1^	Diagnosis		*p* value^2^
		MIS-C diagnosis *n* = 293^1^	Other diagnosis *n* = 1026^1^	
Age at admission (years)	7.0 (2.5, 13.0)	9.0 (5.0, 12.0)	6.0 (2.0, 13.0)	< 0.001

*Sex*				< 0.001
Male	698 (52.9%)	192 (65.5%)	506 (49.3%)	
Female	621 (47.1%)	101 (34.5%)	520 (50.7%)	

*Ethnicity*				0.511
Hispanic or Latino	276 (23.4%)	63 (24.9%)	213 (22.9%)	
Not Hispanic or Latino	906 (76.6%)	190 (75.1%)	716 (77.1%)	
Unknown	137	40	97	

*Race*				0.808
White	626 (59.1%)	136 (57.9%)	490 (59.4%)	
Black/African American	373 (35.2%)	82 (34.9%)	291 (35.3%)	
Asian	40 (3.8%)	11 (4.7%)	29 (3.5%)	
Pacific Islander	12 (1.1%)	4 (1.7%)	8 (1.0%)	
American Indian	9 (0.8%)	2 (0.9%)	7 (0.8%)	
Unknown	259	58	201	

*Patient type*				< 0.001
ED only	305 (23.1%)	6 (2.0%)	299 (29.1%)	
Admitted	1014 (76.9%)	287 (98.0%)	727 (70.9%)	

*Length of stay (days), if admitted*	3.5 (2.0, 6.0)	5.0 (4.0, 7.0)	3.0 (1.7, 5.0)	< 0.001
Unknown	2	0	2	
Received vaccine (1 or 2 doses of the series)	85 (7.4%)	14 (5.9%)	71 (7.8%)	0.308
Unknown	174	55	119	

*Required ICU level care*	321 (24.3%)	161 (54.9%)	160 (15.6%)	< 0.001
Revisit (ED/UC/admitted) related to index visit	114 (8.7%)	14 (4.9%)	100 (9.8%)	0.009
Unknown	15	6	9	

Abbreviations: ED, emergency department; MIS-C, multisystem inflammatory syndrome in children; UC, urgent care.

^1^Median (interquartile range [25th percentile, 75th percentile]), *n* (%).

^2^Mann–Whitney *U* test; Pearson's chi-squared test.

**Table 2:** Median and interquartile ranges of laboratory tests for MIS-C cases and other diagnoses.

Laboratory test	Overall *n* = 1319	MIS-C diagnosis *n* = 293	Other diagnosis *n* = 1026	*p* value^2^	MIS-C vs. other^1^	MIS-C *n*	Other *n*
D-dimer (ng/mL)	1000 (420, 2390)	2334 (1130, 4260)	800 (400, 1656)	< 0.001	+	283	876
Fibrinogen (mg/dL)	479 (370, 575)	513 (412, 585)	452 (341, 575)	< 0.001	+	260	436
LDH (U/L)	384 (269, 658)	363 (268, 574)	410 (269, 669)	0.235		171	334
Albumin (g/dL)	4.0 (3.6, 4.4)	3.5 (3.0, 3.9)	4.1 (3.7, 4.5)	< 0.001	−	292	943
Ferritin (mcg/L)	188 (94, 378)	367 (228, 780)	138 (76, 252)	< 0.001	+	284	634
BNP (pg/mL)	55 (13, 223)	313 (90, 1224)	30 (10, 107)	< 0.001	+	288	801
PTT (s)	31 (27, 35)	30 (27, 35)	31 (27, 35)	0.814		264	544
PT (s)	14 (12, 15)	14 (12, 16)	14 (12, 15)	0.004	+	218	447
INR (nml)	1.2 (1.1, 1.3)	1.2 (1.1, 1.3)	1.1 (1.0, 1.3)	< 0.001	+	263	561
White blood cell count (×10^3^/μL)	9.6 (6.5, 13.7)	9.6 (6.9, 12.5)	9.5 (6.4, 14.1)	0.986		293	983
Absolute neutrophil count (cells/μL)	6200 (3500, 9536)	7300 (4955, 9850)	5735 (3185, 9465)	< 0.001	+	292	974
Absolute lymphocyte count (cells/μL)	1800 (997, 3123)	1100 (700, 1721)	2040 (1118, 3500)	< 0.001	−	290	970
Hemoglobin (g/dL)	12.0 (10.9, 13.0)	11.6 (10.6, 12.7)	12.1 (11.1, 13.1)	< 0.001	−	293	982
Platelet count (×10^3^/μL)	249 (180, 336)	174 (125, 237)	269 (203, 355)	< 0.001	−	293	982
ESR (mm/hr)	36 (14, 59)	44 (26, 66)	32 (11, 59)	< 0.001	+	225	682
CRP (mg/dL)	6.0 (1.3, 13.9)	13.8 (7.5, 21.2)	4.1 (0.8, 10.1)	< 0.001	+	293	967
Procalcitonin (ng/mL)	0.67 (0.17, 2.50)	2.57 (1.03, 8.69)	0.33 (0.12, 1.17)	< 0.001	+	156	372
AST (nml)	36 (25, 54)	41 (27, 59)	35 (24, 53)	0.006	+	263	876
ALT (nml)	20 (13, 38)	31 (17, 55)	18 (12, 30)	< 0.001	+	293	946
IL-6 (pg/mL)	17.5 (5.4, 38.7)	24.5 (11.1, 61.5)	9.6 (4.1, 23.9)	0.002	+	40	43
Triglycerides (mg/dL)	127 (87, 160)	137 (107, 178)	113 (83, 154)	0.017	+	72	118

Abbreviations: ALT, alanine aminotransferase; AST, aspartate aminotransferase; BNP, brain natriuretic peptide; CRP, C-reactive protein; ESR, erythrocyte sedimentation rate; IL, interleukin; INR, international normalized ratio; LDH, lactate dehydrogenase; MIS-C, multisystem inflammatory syndrome in children; PT, prothrombin time; PTT, partial thromboplastin time.

−Symbol indicates that MIS-C had lower value as compared to other diagnosis category.

^1^+Symbol indicates that MIS-C had higher value.

^2^Mann–Whitney *U* test.

**Table 3:** Median and interquartile range of laboratory tests for MIS-C cases and individual other diagnoses.

Laboratory test	MIS-C (*n* = 293)	Acute COVID (*n* = 158)	Acute viral infection(s) (*n* = 352)	Kawasaki disease (*n* = 49)	Rheumatologic condition(s) (*n* = 41)	Sepsis/bacteremia (*n* = 37)
	Median (IQR)	Median (IQR)	Median (IQR)	Median (IQR)	Median (IQR)	Median (IQR)
D-dimer (ng/mL)	2334 (1130, 4260)	691 (400, 1244)	620 (380, 1250)	**1965 (1100, 3148)**	**2670 (1835, 5205)**	**1826 (1018, 3783)**
Fibrinogen (mg/dL)	513 (412, 585)	426 (323, 517)	399 (303, 530)	**496 (404, 589)**	**474 (377, 610)**	**456 (317, 626)**
LDH (U/L)	363 (268, 574)	**401 (245, 811)**	**382 (255, 576)**	**464 (253, 724)**	**605 (279, 866)**	**307 (234, 482)**
Albumin (g/dL)	3.5 (3.0, 3.9)	4.1 (3.8, 4.5)	4.2 (3.8, 4.5)	**3.6 (3.3, 3.8)**	**3.7 (3.3, 4.0)**	**3.6 (3.0, 4.3)**
Ferritin (mcg/L)	367 (228, 780)	167 (78, 326)	119 (71, 219)	169 (119, 293)	**438 (197, 1753)**	176 (82, 396)
BNP (pg/mL)	313 (90, 1224)	10 (10, 58)	28 (10, 97)	59 (20, 285)	23 (10, 126)	80 (20, 357)
PTT (s)	30 (27, 35)	**30 (27, 34)**	32 (28, 36)	**34 (26, 36)**	**31 (27, 37)**	**32 (28, 37)**
PT (s)	14 (12, 16)	14 (12, 15)	13 (12, 15)	**14 (13, 15)**	**14 (13, 16)**	**15 (12, 17)**
INR (nml)	1.2 (1.1, 1.3)	1.1 (1.0, 1.2)	1.1 (1.0, 1.2)	**1.2 (1.1, 1.3)**	**1.2 (1.1, 1.2)**	**1.3 (1.1, 1.5)**
WBC (×10^3^/μL)	9.6 (6.9, 12.5)	7.4 (4.9, 11.3)	**8.9 (6.2, 13.8)**	12.8 (7.1, 16.8)	9.2 (5.0, 13.0)	10.5 (5.9, 15.8)
Absolute neutrophil count (cells/μL)	7300 (4955, 9850)	4500 (2725, 7208)	4690 (2355, 7786)	**7188 (4020, 10, 700)**	**5741 (2600, 9200)**	**7600 (4140, 13, 000)**
Absolute lymphocyte count (cells/μL)	1100 (700, 1721)	1650 (900, 2623)	2610 (1600, 4565)	3080 (1262, 4300)	**1230 (800, 3018)**	**1400 (685, 2280)**
Hemoglobin (g/dL)	11.6 (10.6, 12.7)	12.8 (11.5, 14.2)	12.1 (11.3, 13.0)	10.8 (10.1, 11.6)	**11.5 (10.0, 12.5)**	**11.8 (10.6, 13.1)**
Platelet count (×10^3^/μL)	174 (125, 237)	230 (176, 282)	269 (209, 352)	321 (212, 498)	261 (173, 389)	**198 (126, 291)**
ESR (mm/h)	44 (26, 66)	21 (8, 42)	25 (10, 49)	**48 (31, 75)**	66 (26, 96)	24 (10, 59)
CRP (mg/dL)	13.8 (7.5, 21.2)	2.4 (0.6, 6.2)	2.7 (0.6, 7.2)	9.3 (6.1, 14.6)	5.7 (2.2, 10.4)	**10.0 (4.2, 21.8)**
Procalcitonin (ng/mL)	2.57 (1.03, 8.69)	0.20 (0.06, 0.76)	0.29 (0.14, 1.01)	0.61 (0.35, 2.28)	0.64 (0.10, 1.77)	**3.41 (1.18, 48.86)**
AST (nml)	41 (27, 59)	**37 (25, 59)**	**39 (29, 55)**	**35 (26, 74)**	**54 (27, 78)**	**46 (23, 81)**
ALT (nml)	31 (17, 55)	21 (15, 46)	17 (13, 27)	**23 (14, 54)**	**21 (14, 53)**	**26 (14, 62)**
IL-6 (pg/mL)	24.5 (11.1, 61.5)	6.2 (3.6, 10.8)	5.1 (0.0, 13.6)	**12.6 (3.1, 54.7)**	**38.1 (6.3, 40.3)**	**20.1 (12.9209.6)**
Triglycerides (mg/dL)	137 (107, 178)	87 (68, 152)	**138 (87, 140)**	**122 (108, 164)**	**146 (102, 247)**	**101 (87, 375)**

*Note:* Bolded cells indicate an IQR that is not significantly different than the IQR for MIS-C (*p* > 0.05), Mann–Whitney *U* test.

Abbreviations: ALT, alanine aminotransferase; AST, aspartate aminotransferase; BNP, brain natriuretic peptide; CRP, C-reactive protein; ESR, erythrocyte sedimentation rate; IL, interleukin; INR, international normalized ratio; IQR, interquartile range; LDH, lactate dehydrogenase; MIS-C, multisystem inflammatory syndrome in children; PT, prothrombin time; PTT, partial thromboplastin time.

## Data Availability

Data are available on request from the authors.

## Supporting Information

The file named MIS-C Supporting Table for EMI contains Supporting Table 1, Elevated Troponin Levels in Patients Evaluated for MIS-C.
