# Supplementary material for: Normative Laboratory Value Ranges in Pediatric Patients Who Underwent Evaluation for MIS-C
Source: Emerg Med Int. 2025 Nov 25;2025:2660909. doi: 10.1155/emmi/2660909 (PMC12672071; doi:10.1155/emmi/2660909)
Supplement: Supporting Information — Additional supporting information can be found online in the Supporting Information section. [file 2660909.f1.docx]

| **Diagnosis** | **Troponin >.02** | **Total # Tested** | v. MIS-C |
| --- | --- | --- | --- |
| MIS-C | 108 (37.5%) | 288 | <.001 |
| All Other Diagnoses | 68 (8.3%) | 820 |  |
| Acute COVID | 9 (8.6%) | 105 | <.001 |
| Acute Viral Infections(s) | 17 (6.0%) | 283 | <.001 |
| Kawasaki Disease | 3 (7.0%) | 43 | <.001 |
| Sepsis and/or Bacteremia | 5 (14.7%) | 34 | .008 |
| Fever of Unknown Origin | 3 (5.7%) | 53 | <.001 |
| Pyelonephritis | 5 (6.5%) | 77 | <.001 |
| Appendicitis | 0 (.0%) | 15 | .003 |
| Other Bacterial Infection | 1 (2.9%) | 34 | <.001 |
| Herpangina | 0 (.0%) | 11 | .009 |
| Gastroenteritis | 6 (12.0%) | 50 | <.001 |
| Pneumonia | 2 (3.9%) | 51 | <.001 |
| DRESS | 0 (.0%) | 9 | .029 |
| Rheumatologic Condition(s) | 5 (16.7%) | 30 | .023 |
| EVALI | 0 (.0%) | 9 | .029 |
| Myocarditis | 8 (80.0%) | 10 | .016 |
| Other | 21 (14.2%) | 148 | <.001 |

**Supplemental Table 1: Elevated Troponin Levels in Patients Evaluated for MIS-C**

DRESS – drug reaction with eosinophilia and systemic symptoms; EVALI – e-cigarette or vaping use-associated lung injury
